# Grapefruit-derived nanovectors deliver miR-18a for treatment of liver metastasis of colon cancer by induction of M1 macrophages

**DOI:** 10.18632/oncotarget.8361

**Published:** 2016-03-25

**Authors:** Yun Teng, Jingyao Mu, Xin Hu, Abhilash Samykutty, Xiaoying Zhuang, Zhongbin Deng, Lifeng Zhang, Pengxiao Cao, Jun Yan, Donald Miller, Huang-Ge Zhang

**Affiliations:** ^1^ Robley Rex VA Medical Center, Louisville, KY 40206, USA; ^2^ James Graham Brown Cancer Center, University of Louisville, Louisville, KY 40202, USA; ^3^ Department of Microbiology and Immunology, University of Louisville, Louisville, KY 40202, USA; ^4^ Program in Biostatistics, Bioinformatics and Systems Biology, The University of Texas Graduate School of Biomedical Sciences at Houston, Houston, TX 77030, USA; ^5^ Department of Genomic Medicine, The University of Texas MD Anderson Cancer Center, Houston, TX 77030, USA

**Keywords:** miR-18a, M1 Kupffer cells, grapefruit-derived nanovector, IRF2, liver metastasis of colon cancer

## Abstract

Liver metastasis accounts for many of the cancer deaths in patients. Effective treatment for metastatic liver tumors is not available. Here, we provide evidence for the role of miR-18a in the induction of liver M1 (F4/80^+^interferon gamma (IFNγ)^+^IL-12^+^) macrophages. We found that miR-18a encapsulated in grapefruit-derived nanovector (GNV) mediated inhibition of liver metastasis that is dependent upon the induction of M1 (F4/80^+^IFNγ^+^IL-12^+^) macrophages; depletion of macrophages eliminated its anti-metastasis effect. Furthermore, the miR-18a mediated induction of macrophage IFNγ by targeting IRF2 is required for subsequent induction of IL-12. IL-12 then activates natural killer (NK) and natural killer T (NKT) cells for inhibition of liver metastasis of colon cancer. This conclusion is supported by the fact that knockout of IFNγ eliminates miR-18a mediated induction of IL-12, miR-18a treatment has an anti-metastatic effects in T cell deficient mice but there is no anti-metastatic effect on NK and NKT deficient mice. Co-delivery of miR-18a and siRNA IL-12 to macrophages did not result in activation of co-cultured NK and NKT cells. Taken together our results indicate that miR-18a can act as an inhibitor for liver metastasis through induction of M1 macrophages.

## INTRODUCTION

Metastasis accounts for the majority of cancer deaths. The liver is a frequent site of metastasis of many different types of cancer, including colon cancer. Liver macrophages (Kupffer cells; KCs) play a crucial role in the pathogenesis of liver tumor metastasis and are a major component of the microenvironment of primary and metastatic liver tumors. Direct and indirect activation of KCs results in the production of factors and cytokines capable of facilitating both anti-tumor [1–5] and pro-tumor effects [6–8]. More importantly, Kupffer cells are situated in the hepatic sinusoids to encounter circulating T cells, as well as natural killer (NK) and natural killer T (NKT) cells, and modulate activity of these lymphocytes. Interaction with these immune cell populations is required to develop the full potential of KCs to mediate anti-tumor immunity [9–12]. Therefore, targeted delivery of therapeutic agents to liver KCs could enhance anti-tumor immune functions.

Evidence is provided that liver macrophages can make M1 or M2 responses. M1 and M2 macrophages promote Th1 and Th2 responses, respectively. M2 macrophages are a major component of the leukocyte infiltrate of tumors. M2 macrophages suppress NK, NKT, and T-cell activation and proliferation by releasing transforming growth factor beta (TGF-β) [13–19]. Moreover, they have an interleukin (IL)-12^low^ phenotype, characteristic of M2 cells. By expressing properties of polarized M2 cells, M2 participate in circuits that regulate tumor growth and progression, adaptive immunity, stroma formation and angiogenesis. This raises the possibility that the molecules and cells involved might represent novel and valuable therapeutic targets. As for M1 macrophages, these macrophages produce IL-12 [16, 20–24] to promote tumoricidal responses. The mechanisms governing macrophage polarization are unclear.

MicroRNAs (miRNAs) are a class of small, non-coding RNAs that post-transcriptionally control the translation and stability of mRNAs. Hundreds of miRNAs are known to have dysregulated expression in cancer [25–30]. Studies evaluating their biological and molecular roles and their potential therapeutic applications are emerging. The levels of miRNAs expressed in myeloid cells have effects on the polarization of M1 versus M2 macrophages [31–36]. Targeted delivery of miRNAs to macrophages as an alternative strategy for treatment of cancer by induction of M1 macrophages has not been fully developed.

MiR-18a, an important member of miR-17–92 family, has been shown various effects on different tumors. It was reported that miR-18a could act as a tumor suppressor. Our previous study published showed that miR-18a suppresses colon tumor growth by targeting β-catenin expressed in the colon tumor cells. The effects of miR-18a on the polarization of M1 versus M2 macrophages have not been reported. We attempted to predict the potential target genes of miR-18a through applying a bioinformatics analysis method (TargetScan). We found *Irf2*, a theoretical target gene of miR-18a with the specific binding site in the 3′-UTR sequence. IL-12 is dysregulated in macrophages from Irf2 knockout mice. This finding led us to choose miR-18a as an example to test whether a grapefruit-derived nanovector (GNV) based delivery system can be used for targeted delivery of therapeutic miRNA to liver macrophages and treat liver metastasis.

## RESULTS

### Optimization of efficiency of OGNVs for encapsulating RNA

We first tested whether the efficiency of OGNVs for encapsulating RNA in general can be increased by Ultraviolet (UV) cross-linking lipids extracted from grapefruit nanoparticles with RNAs extracted from CT26 cells. Lipids extracted from sucrose gradient purified grapefruit nanoparticles (Supplementary Figure S1) and cellular RNA were mixed and exposed to different doses of UV light (254 nm) using a Spectrolinker. The results showed that lipids pre-exposed to UV radiation at 250 millijoules seconds per cm^2^ (mJ/cm^2^) and 500 mJ/cm^2^ reassembled into OGNVs with a diameter of 110.7 ± 22.5 nm (means ± standard error of the mean (SEM)) and 120.6 ± 15.7 nm, respectively (Supplementary Figure S2A). Both doses of UV radiation resulted in an increased efficiency of encapsulation for RNA from 5.5 ± 2.2% to 28.2 ± 4.8% and 30.6 ± 4.5%, respectively (Supplementary Figure S2B). However, further increasing the dose of UV (1,000 mJ/cm^2^ and 2,000 mJ/cm^2^) resulted in decreasing the encapsulation efficiency of RNA.

Next, we tested whether neutralizing negative charges of the RNAs might further enhance the efficiency of encapsulation of RNA in OGNVs. OGNVs were assembled by sonication of grapefruit nanoparticle-derived lipids with RNA pre-dissolved in H_2_O, phosphate buffered saline (PBS, pH 7.4), and 155 mM sodium chloride (NaCl). Using 155 mM NaCl caused a 4.3-fold and 3.9-fold more efficient encapsulation of RNA than H_2_O and PBS, respectively (Supplementary Figure S2C). Furthermore, an additive effect was observed when NaCl was combined with UV radiation (Supplementary Figure S2C). The efficiency of encapsulation of RNA when placed in NaCl and exposed to UV radiation was increased markedly in comparison with H_2_O combined with UV exposure (49.6% vs 27.32%) or PBS combined with UV exposure (49.6% vs 28.62%). Collectively, the combination of UV radiation (500 mJ/cm^2^) and NaCl (155 mM) provides optimal conditions for enhancing RNA encapsulation efficiency in OGNVs. Henceforth we refer to the nanovectors made under these conditions as optimized-GNVs (OGNVs).

To determine whether UV radiation and NaCl have an effect on the functional characteristics of RNA encapsulated in OGNVs, we evaluated the size (Supplementary Figure S3A, S3B) and potential distribution (Supplementary Figure S3C) of OGNVs using a Zetasizer Nano ZS. With UV radiation, the average diameter of the OGNVs was 156 ± 33 nm in NaCl, in comparison with 125 ± 22 nm in H_2_O, and 188 ± 28 nm in PBS. Zeta potential analysis revealed that OGNVs in H_2_O displayed a negative charge of -47.6 ± -9.61 mV. A NaCl concentration of 155 nM remarkably neutralized the charge of OGNVs to −3.4 ± 1.7 mV (*p* < 0.01), but PBS did not change the charge of OGNVs. Taken together, these data suggest that NaCl treatment of RNA not only increases encapsulation in OGNVs but alters the charge of OGNVs from strongly negative to weakly negative without dramatically affecting the size of the OGNVs.

To further determine whether RNA has been encapsulated in the OGNVs or is located on the surface of OGNVs, OGNVs carrying Exo-GLOW (red) labeled RNA were digested with ribonucleases (RNase). Fluorescence analysis using confocal microscopy revealed RNA was still co-localized with OGNVs after RNase treatment (Supplementary Figure S3D, S3E). Furthermore, without detergent extraction, OGNV RNA was resistant to RNase digestion when OGNVs were kept at 4°C for 7 days; whereas after extraction from OGNVs, the RNA without encapsulation in OGNVs was degraded by RNase (Supplementary Figure S4). Collectively, these results suggest that potentially therapeutic RNA can be encapsulated into OGNVs. Following this we determined whether UV treatment of OGNVs has an effect on the biological activity of encapsulated RNA. To address this concern, 20 μg of luciferase siRNA encapsulated in the OGNVs was transfected into U-87 MG-luc, a luciferase positive glioblastoma cell line which stably expresses the firefly luciferase gene. Assessment of luciferase activity with the Dual-Luciferase Reporter Assay System revealed that a similar activity of luciferase siRNA was demonstrated in the U-87 MG-luc cells transfected with OGNVs (40%) and polyethylenimine (PEI) (45%) (Supplementary Figure S3F), a commercial RNA delivery agent.

### miR-18a encapsulated in OGNVs (OGNVs-miR-18a) induces M1 Kupffer cells

**Figure 1:**
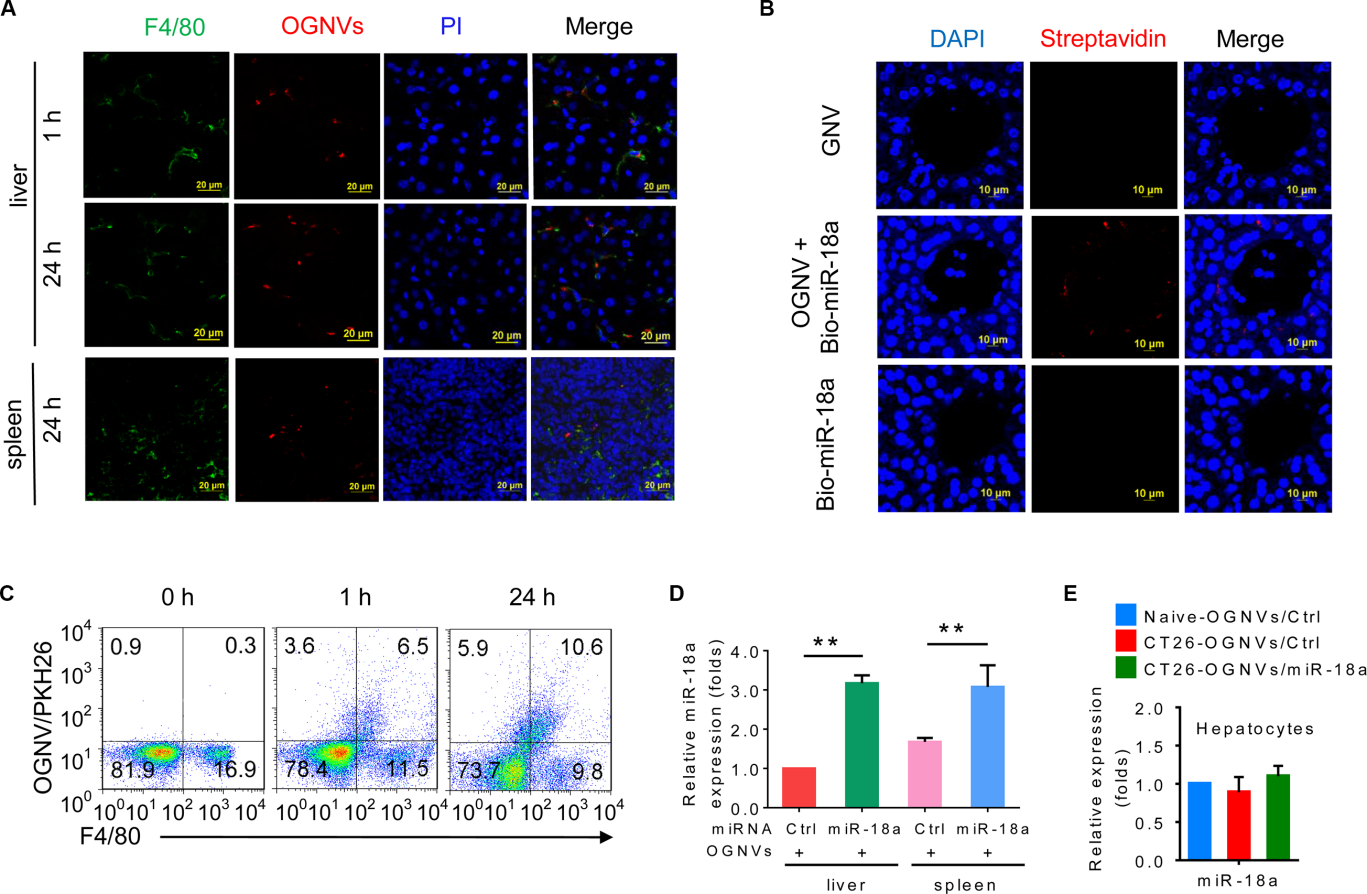
OGNV-mediated delivery of miRNA is taken up by mouse Kupffer cells *in vivo* (**A**) PKH26-labeled (red) OGNVs located in liver Kupffer cells (F4/80^+^, green), not in spleen macrophages (F4/80^+^, green) from BALB/c mice are visualized with confocal microscopy, assessed 1 h and 24 h after intravenous injection. (**B**) Analysis of Alexa Fluor fluorescent streptavidin conjugates with confocal microscope, assessed 24 h after intravenous injection of OGNVs alone, OGNVs with biotin-conjugated miR-18a (bio-miR-18a), or bio-miR-18a alone. (**C**) Frequency of F4/80^+^ cells and PKH26-labled OGNVs in the liver from BALB/c mice assessed using flow cytometry. Numbers in quadrants indicate percent cells in each. (**D**) Quantification of miR-18a level in leukocytes from BALB/c mouse liver and spleen assessed 24 h after intravenous injection of OGNVs with miR-18a by quantitative real-time PCR (qPCR). *P < 0.05 and **P < 0.01 (two-tailed *t*-test). Data are representative of three independent experiments (error bars, S.E.M.). (**E**) Expression of miR-18a in hepatocytes from naive BALB/c mice, CT26 liver metastasis mice with OGNVs/Ctrl or OGNVs/miR-18a treatment assessed by quantitative real-time PCR (qPCR).

Liver KCs (Figure 1A–1D) but not hepatocytes (Figure 1E) take up OGNVs carrying miR-18a after a tail vein injection. KCs represent 80–90% of all tissue macrophages in the entire body [37], play a major role in the capture and clearance of foreign material, are important antigen presenting cells (APCs), and express MHC I, MHC II and costimulatory molecules needed for activation of immune cells. Collectively, these features of liver KCs prompted us to test whether GNVs can be used as a vehicle for delivery of therapeutic agents for treatment of liver related disease through the mechanism of immunomodulation of Kupffer cells. Therefore, we set out to determine whether miR-18a delivered by OGNVs has a biological effect on liver metastasis of colon cancer as an example.

**Figure 2:**
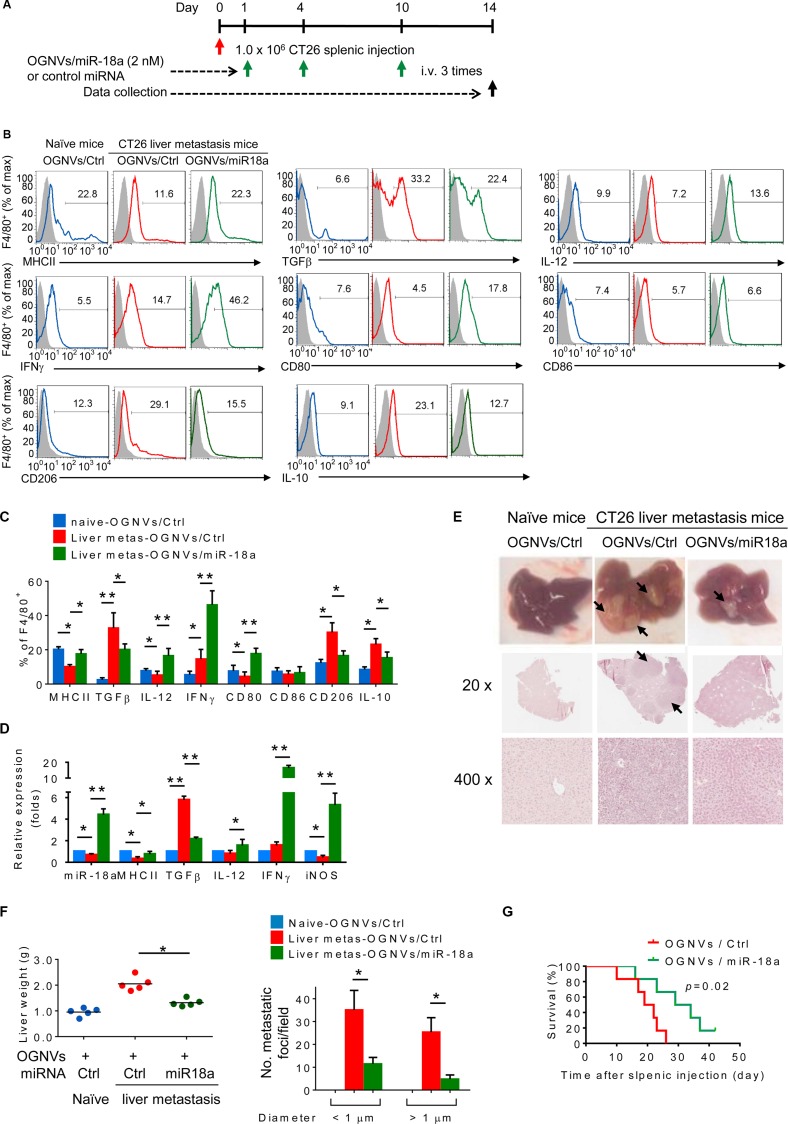

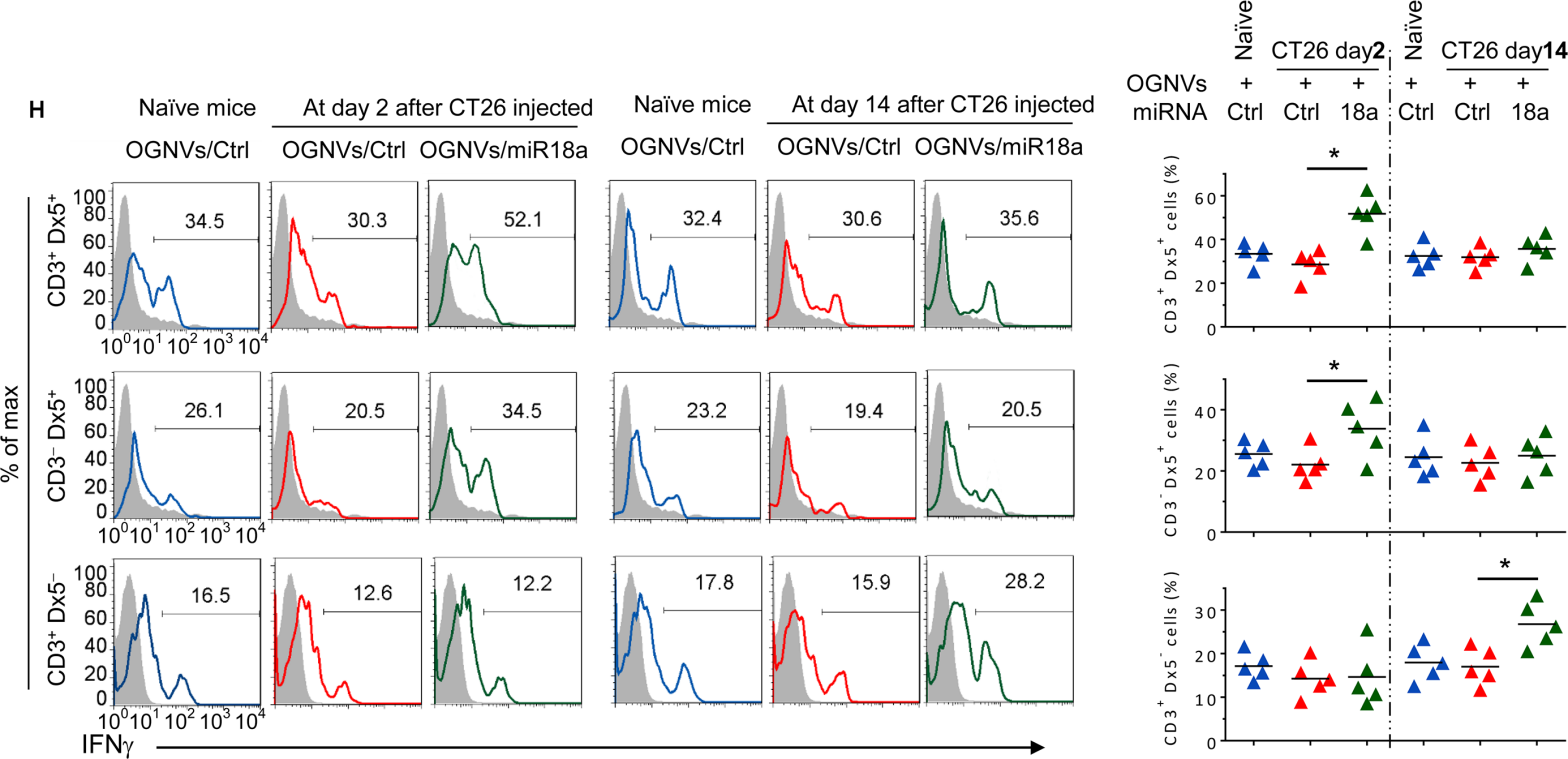
miR-18a encapsulated in OGNVs inhibits liver metastasis of colon cancer and induces Kupffer cell polarization into M1 (**A**) Schematic representation of the treatment schedule. All groups of mice were euthanized 14 days after the intra-splenic tumor inoculation, and tumor specimens were obtained for analysis. (**B**) Frequency of MHCII, TGFβ, IL-12, IFNγ, CD80, CD86, CD206, and IL-10 positive cells in liver F4/80^+^ cells from naive BALB/c mice, CT26 liver metastasis mice treated with OGNVs packing control miRNA (OGNVs/Ctrl) or OGNVs packing miR-18a (OGNVs/miR-18a) assessed by flow cytometry. (**C**) The histogram shows the quantification of results at (b). (**D**) Expression of mature miR-18a, MHCII, TGFβ, IL-12, IFNγ, and iNOS in liver F4/80^+^ cells was assessed by qPCR. (**E**) Representative livers (up) and representative hematoxylin and eosin (H & E)-stained sections of livers (middle, 20×; bottom, 400× magnification). (**F**) Liver weight (left) and liver metastatic nodule number and size (right). (**G**) Survival of mice after intra-splenic injection of CT26 cells. (**H**) Frequency of IFNγ^+^ cells in liver CD3^+^Dx5^+^ (NKT) cells, CD3^−^Dx5^+^ (NK) cells, and CD3^+^ Dx5^−^ (T) cells. Right, quantification of results; each symbol represents an individual mouse. *P < 0.05 (two-tailed *t*-test). Data are representative of three independent experiments (error bars, S.E.M.).

OGNV-miR18a treatment, as described in Figure 2A, led to an increase in the percentages of F4/80^+^ major histocompatibility complex (MHC)II^+^, F4/80^+^IL-12^+^ (M1), F4/80^+^interferon gamma (IFNγ)^+^ and F4/80^+^CD80^+^ cells (Figure 2B). This increase is specific since the percentages of F4/80^+^CD86^+^ cells present in the liver of tumor bearing mice treated with OGNVs/Ctrl alone were no different from those treated with OGNVs-miR18a (Figure 2B). It is well-known that M1 macrophages promote anti-tumor activity whereas M2 macrophages promote tumor progression. We further assessed the M1 versus M2 cytokine expressions in liver F4/80^+^ cells. miR-18a treatment led to increasing percentages of F4/80^+^IFNγ^+^, F4/80^+^IL-12^+^, F4/80^+^CD80^+^, and decreasing percentages of F4/80^+^ transforming growth factor beta (TGFβ)^+^, F4/80^+^CD206^+^ and F4/80^+^ IL-10^+^ detected in the liver metastatic tumor bearing mice (Figure 2B). This result was also supported by the data from quantitative analysis of the proteins expressed on FACS sorted F4/80 KCs (Figure 2C). Consistent with flow cytometry results, OGNV-miR18a treatment dramatically increased the level of genes encoding IFNγ, IL-12, CD80, inducible nitric oxide synthase (iNOS), and decreased TGFβ expressed in F4/80 KCs isolated from metastatic liver (Figure 2D). Collectively, miR-18a treatment promoted induction of M1 macrophages (F4/80^+^IFNγ^+^ and F4/80^+^IL-12^+^) with upregulated co-stimulatory factors such as CD80, and iNOS while inhibiting M2 macrophages (F4/80^+^TGFβ^+^, F4/80^+^IL-10^+^) in the liver of metastatic colon tumor bearing mice.

The inhibition of liver metastatic tumor growth in CT26 tumor bearing mice treated with OGNV-miR18a was also demonstrated. On day 14 after an intra-splenic injection of CT26 colon tumor cells, the number and size of tumor nodules in the liver of mice treated with vehicle were significantly increased in comparison with mice treated with OGNV-miR18a (Figure 2E). This conclusion is also supported by the fact that there were fewer liver tumor foci, the liver weighed less in OGNV-miR18a treated mice (Figure 2F) and these mice had a significantly prolonged survival (Figure 2G).

**Figure 3:**
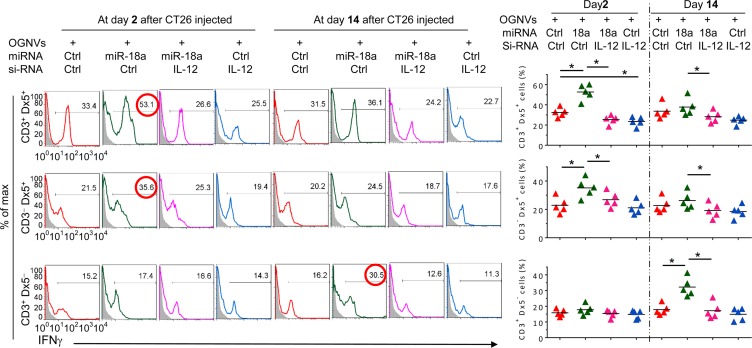
Induction of IFNγ^+^NK and IFNγ^+^NKT by OGNVs-miR-18a Frequency of IFNγ^+^ cells in liver CD3^+^Dx5^+^ (NKT) cells, CD3^−^Dx5^+^ (NK) cells, and CD3^+^Dx5^−^ (T) cells from CT26 liver metastasis mice treated with OGNVs-Ctrl, OGNVs-miR-18a with/without IL-12 siRNA knockdown assessed by flow cytometry (Left); Right, quantification of FACS analyzed results; each symbol represents an individual mouse.

The induction of M1 macrophages promotes activation of NK, NKT and T cells. The data generated from FACS analysis indicated that at day 2 after OGNV-miR-18a treatment, both IFNγ^+^ NKT (CD3^+^DX5^+^) and IFNγ^+^NK (CD3^−^DX5^+^) but not T(CD3^+^DX5^−^) cells were significantly induced; whereas, on day 14 induction of IFNγ^+^ CD3^+^T cells was dominant (Figure 2H). To further demonstrate the role of macrophage-derived IL-12 induction of IFNγ^+^NK and IFNγ^+^NKT, mice treated with OGNVs co-encapsulating miR-18a and IL-12 siRNA but not encapsulating IL-12 siRNA alone resulted in significant reduction of liver IFNγ^+^ NK and IFNγ^+^NKT, but had no effect on IFNγ^+^CD3^+^DX5^−^ T cells (Figure 3). Consistent with *in vivo* results, neutralizing IL-12 in the supernatants of miR-18a pre-transfected IL-12^+^ RAW264.7 macrophage-like cells (Supplementary Figure S5) co-cultured with primary spleen NKT cells led to a significant reduction of IFNγ expressed in the NKT cells (Supplementary Figure S5). Collectively, these results suggest that F4/80^+^IL-12^+^ cells induced by OGNV-miR-18a plays a crucial role in the inhibition of liver metastasis of colon cancer.

### Liver macrophages play a dominate role in inhibition of colon tumor metastasis in the liver

**Figure 4:**
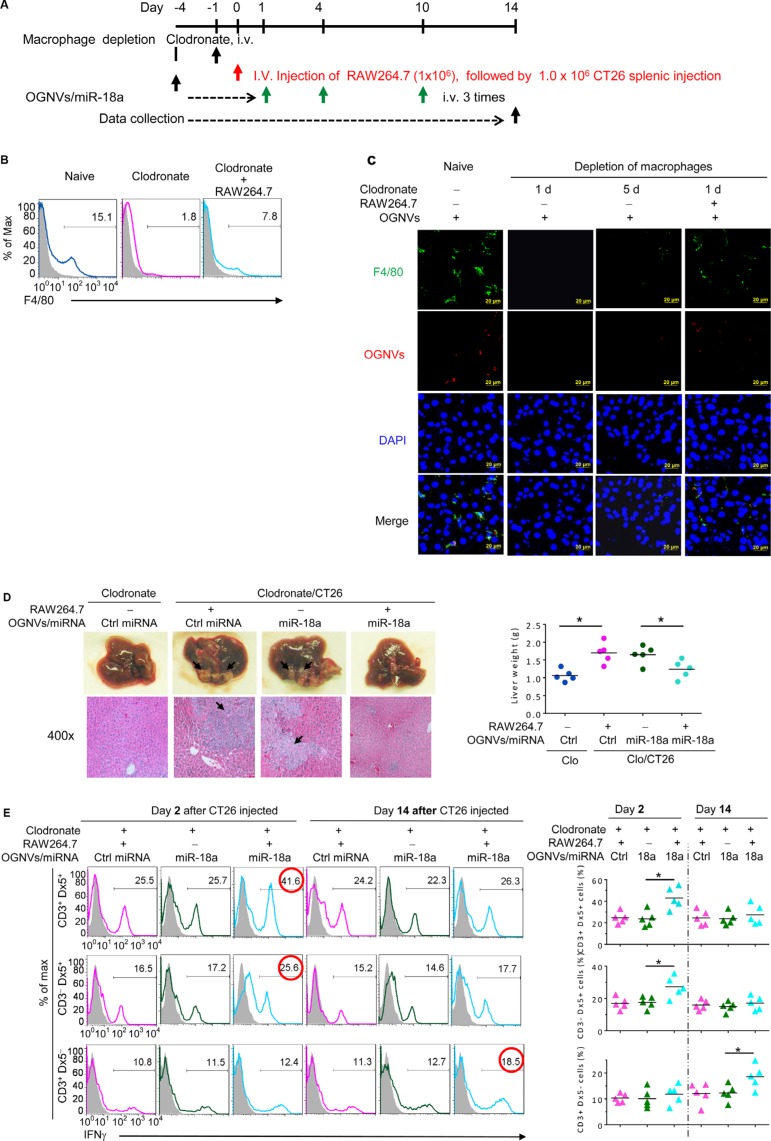
Depletion of macrophages restricted the response of miR-18a against liver metastasis (**A**) Schematic representation of treatment schedule. All groups of mice were euthanized 14 days after the intra-splenic tumor injection, and tumor specimens were obtained for analysis. (**B**) Frequency of F4/80^+^ cells in liver leukocytes from clodronate treated (110 mg/kg) mice, with or without RAW264.7 cells assessed by flow cytometry. (**C**) PKH26-labeled (red) OGNVs located in liver Kupffer cells (F4/80^+^, green) were visualized with confocal microscopy at 1 d and 5 d after administer of clodronate. Data are representative of three independent experiments. (**D**) Representative for the treatment effect on liver metastasis (left, upper panel) and hematoxylin and eosin (H & E)-stained liver sections (left bottom panel) from Kupffer cell depleted mice with or without RAW264.7 cells adoptively transferred, Right; Liver weight. (**E**) Frequency of IFNγ positive cells (left panel) in liver CD3^+^Dx5^+^ (NKT) cells, CD3^−^Dx5^+^ (NK) cells, and CD3^+^Dx5^−^ (T) cells from OGNVs/Ctrl miRNA and OGNVs/miR-18a treated mice with/without macrophages pre-depleted. The percentages of positive NK, NKT, and T cells are shown (right panel); each symbol represents an individual mouse. *P < 0.05 (two-tailed *t*-test). Data are representative of three independent experiments (error bars, S.E.M).

To identify whether the anti-tumor activity of miR-18a was directly mediated by liver macrophages, mice were repeatedly treated with clodronate liposome as described in Figure 4A to deplete macrophages before an intra-splenic injection of CT26 cells. Depletion of macrophages (Figure 4B, 4C) abolished the anti-tumor activity of miR-18a, and the miR-18a-mediated anti-tumor activity was restored by adoptive transfer of macrophage-like RAW264.7 cells (Figure 4D). This conclusion is also supported by the significant induction of liver IFNγ^+^NKT and IFNγ^+^NK cells at day 2 and IFNγ^+^ CD3^+^T cells on day 14 after RAW264.7 cells were adoptively transferred into macrophage depleted mice (Figure 4E).

### miR-18a-mediated inhibition of the growth of liver metastasis of colon tumor cells is IFNγ dependent

**Figure 5:**
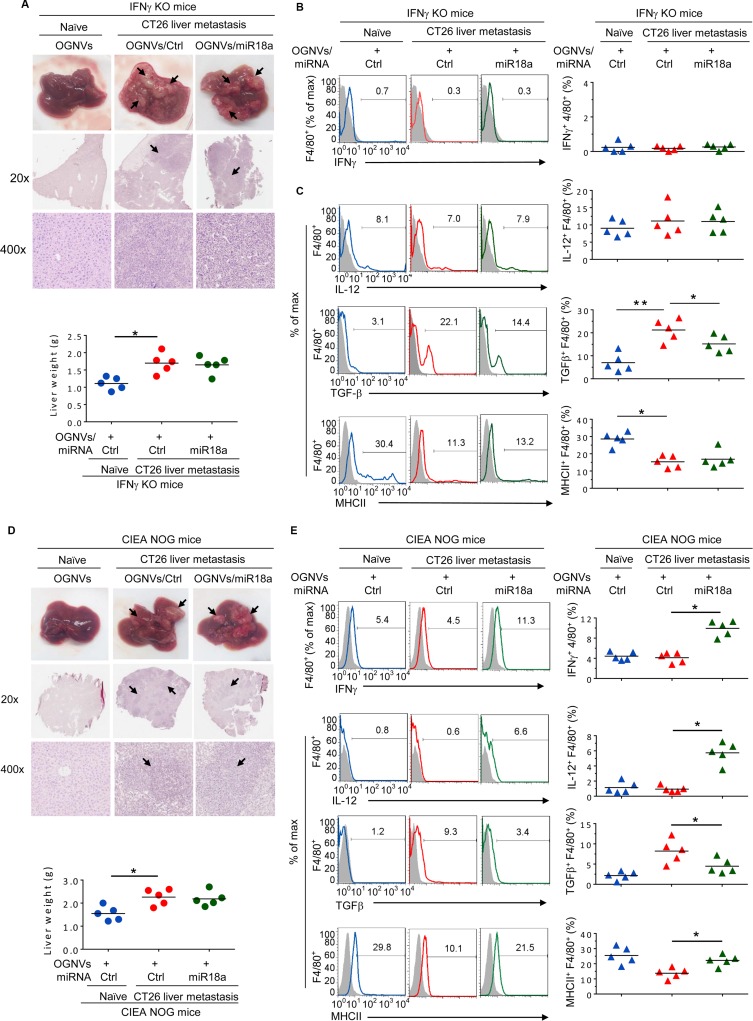

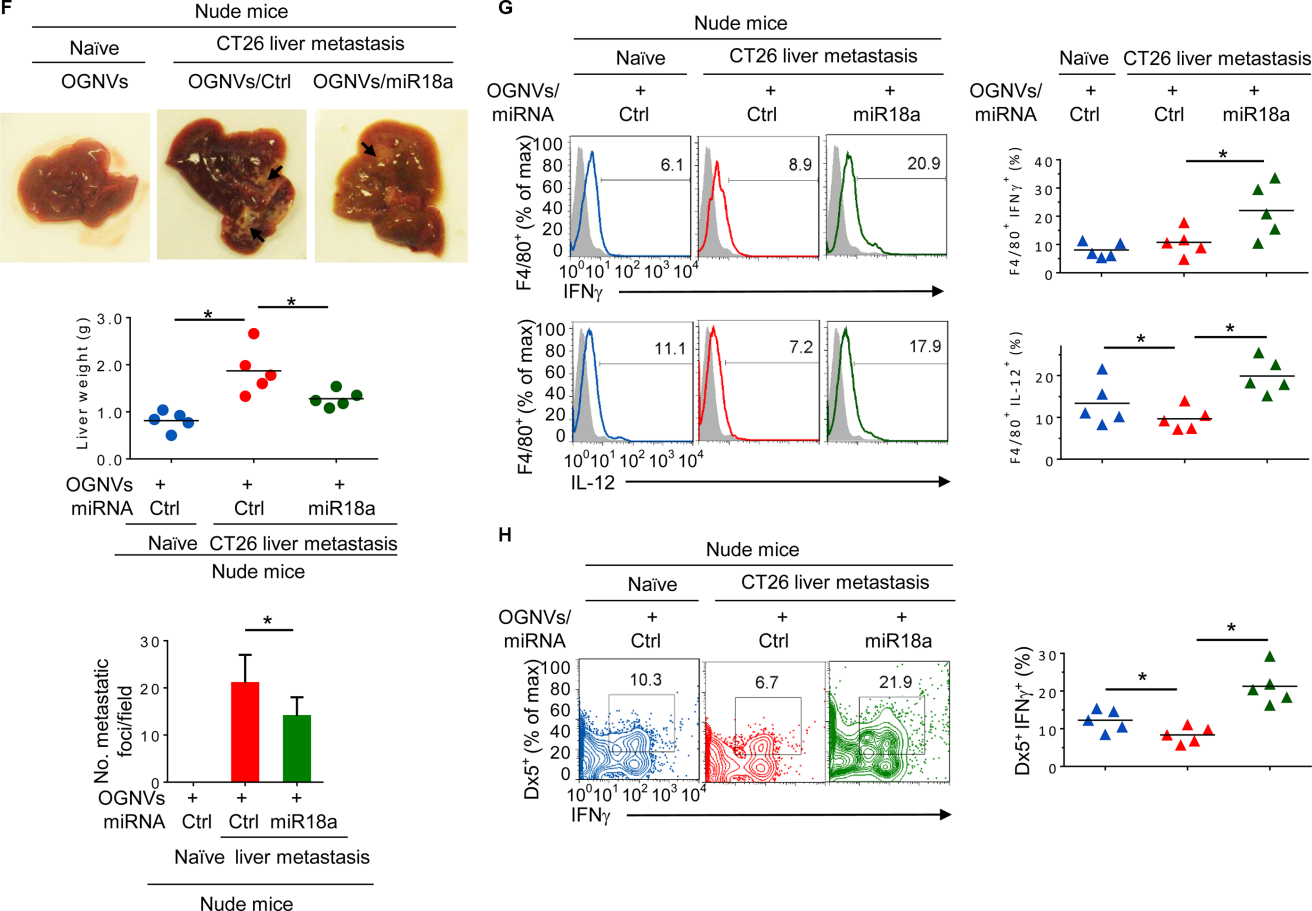
miR-18a mediated inhibition of the growth of liver metastasis of colon tumor cells is IFNγ dependent (**A**) Representative livers (up) (metastatic nodules shown by arrows) and H & E-stained sections of livers (middle, 20×; bottom, 400× magnification) from IFNγ knockout (KO) naïve mice. Liver weight of IFNγ KO mice (bottom). (**B**) Frequency of IFNγ^+^F4/80^+^ cells in liver from IFNγ KO mice (Naïve) and CT26 liver metastatic mice was assessed by flow cytometry. The percentages of IFNγ+F4/80+ cells in liver and each symbol represents the FACS data from individual mice (right panel). (**C**) Frequency of IL-12, TGFβ, MHCII positive cells in liver F4/80^+^ cells from IFNγ KO mice was assessed by flow cytometry. The percentages of double positively stained cells from treated mice are presented, and each symbol represents the FACS data from individual mice (right panel). (**D**) Representative livers (upper) and H&E-stained sections of livers (middle, 20x; bottom, 400x magnification) from NOG mice treated as labeled in the figure are shown (upper panel), and liver weight of NOG mice treated as labeled in the figure is indicated (bottom panel). (**E**) Frequency of liver F4/80^+^IFNγ^+^, F4/80^+^IL-12^+^, F4/80^+^MHCII^+^ and F4/80^+^TGFβ^+^ cells from NOG mice treated as indicated in the labels of figure 5e. Percent double positive cells (right panels). (**F**) Representative livers (up) from athymic nude mice. Middle: liver weight. Bottom: quantification of liver metastatic foci. (**G**) Frequency of IFNγ and IL-12 positive cells in liver F4/80^+^ KC cells. (**H**) Frequency of IFNγ positive cells in liver Dx5^+^NK cells. *P < 0.05 (two-tailed *t*-test). Data are representative of three independent experiments (error bars, S.E.M.).

To determine whether the effect of miR-18a against liver metastasis of colon cancer results from induction of KC IFNγ, CT26 colon carcinoma cells were intra-splenic injected into IFNγ knock out (KO) mice. On day 14 after tumor cell inoculation, OGNVs/miR-18a treatment showed no evidence of inhibiting tumor growth in IFNγ KO mice. Mice treated with OGNVs/control (Ctrl)-miRNA alone and OGNVs/miR18a were similar in liver size and weight (Figure 5A). The H & E stained sections of liver from both groups displayed similar pathology of liver metastasis (Figure 5A). As expected, IFNγ expression was not found on leukocytes or F4/80 cells from the livers in IFNγ KO mice (Figure 5B). Evidence for the effect of miR-18a on induction of F4/80^+^IL-12^+^ was not obtained in IFNγ KO mice although the expression of TGFβ was still repressed by miR-18a (Figure 5C). Collectively, these results indicate that KC IFNγ is an upstream cytokine of IL12 for miR-18a mediated induction of M1 macrophages. KC IFNγ is required for miR-18a-mediated induction of IL-12. Induction of macrophage IL-12 further enhances activation of NK and NKT cells at positive feed-back manner. To further clarify the role of NK, NKT and T cells on the inhibition of tumor metastasis caused by miR-18a, NOG mice which are deficient for NK, NKT, and T cells were challenged with CT26 tumor cells using the identical protocol described for induction of liver metastasis of colon cancer in a wild-type BALB/c mouse model (Figure 2). As expected, multi-administration of OGNVs-miR-18a did not lead to inhibition of tumor metastasis in the NOG mice (Figure 5D) although F4/80^+^IFNγ^+^, F4/80^+^IL-12^+^ and F4/80^+^MHCII^+^ cells (Figure 5E) were still induced. The fact that the frequency of CD3^+^ and Dx5^+^ cells were undetectable in naïve or tumor bearing NOG mice (Supplementary Figure S6) regardless of treatment supports the idea that NK, NKT, or T cells are effector cells responsible for inhibition of liver metastasis of colon cancer cells. In contrast, the data generated from nude mice (Figure 5F) which have both NK and NKT cell activity suggest that NK and NKT cells play a critical role in the inhibition of tumor metastasis caused by miR-18a. The effects of miR-18a on induction on IFNγ^+^IL-12^+^KCs (Figure 5G) and IFNγ^+^NK^+^ cells (Figure 5H) has no impact in T cell deficient nude mice. In combination with data generated from macrophage depletion, IFNγ KO mice and NOG and nude mice, these data suggest that miR-18a delivered by OGNVs initially induces expression of IFNγ in macrophages, which is required for induction of macrophage IL-12. Subsequently, macrophage IL-12 amplifies the miR-18a-mediated anti-tumor activity by activation of liver NK and NKT cells in an IFNγ dependent manner.

### miR-18a suppresses liver metastasis of colon cancer triggered by directly targeting IRF2

**Figure 6:**
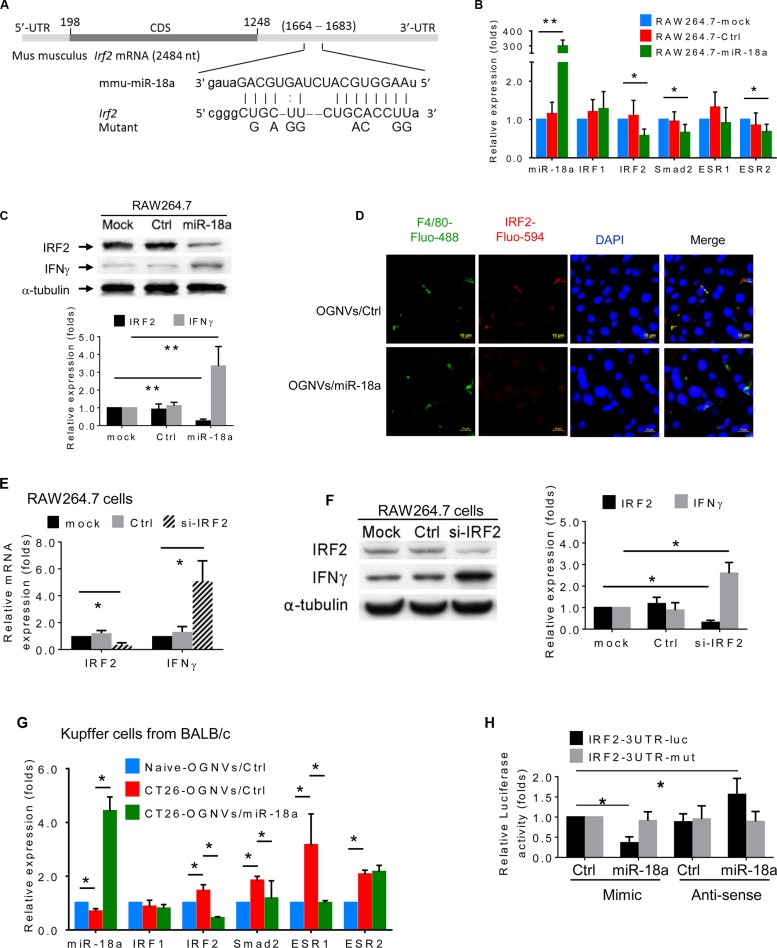
miR-18a suppresses liver metastasis of colon cancer triggered by direct targeting of *Irf2* expressed in Kupffer cells (**A**) Schematic diagram of the putative binding sites of miR-18a in the wide type (WT) IRF2 3′ untranslated regions (UTR). The miR-18a seed matches in the IRF2 3′UTR are mutated at the positions as indicated. CDS, coding sequence. (**B**) Expression of miR-18a and potential miR-18a targeted genes in macrophages-like RAW264.7 cells was analyzed by real-time PCR. (**C**) Expression of candidate miRN-18a target gene IRF2 and IFNγ in macrophage RAW264.7 cells assessed by western blotting. (**D**) IRF2 (red) expression in liver of CT26/OGNVs and CT26/OGNVs/miR-18a treated mice, visualized with a confocal microscopy. Data are representative of three independent experiments (*n* = 5). (**E**) Evaluation of IRF2 and IFNγ level in macrophage-like RAW264.7 cells assessed by qPCR, 72 h after transfection of IRF2 siRNA (si-IRF2) or control (Ctrl) siRNA. (**F**) Expression of IRF2 and IFNγ in aliquots of macrophage-like RAW264.7 cells assessed by western blotting (left), quantification of results (right). (**G**) Expression of miR-18a and candidate miR-18a target genes in liver F4/80^+^ cells sorted by FACS and assessed by real-time PCR, following intravenous administration of OGNVs/miR-18a mimic and OGNVs/control miRNA. (**H**) Luciferase activity assays of WT and mutated Irf2 3′UTR luciferase reporters after co-transfection with miR-18a mimic, miRNA mimic control, miR-18a anti-sense RNA (AS-miR-18a), or miRNA anti-sense negative control RNA in RAW264.7 cells. The luciferase activity of each sample was normalized to the Renilla luciferase activity. The normalized luciferase activity of transfected control mimic miRNA was set as relative luciferase activity of 1. Error bars represent S.E.M. Each data point was measured in triplicate.

Given the profound anti-colon tumor metastasis effect of miR-18a delivered by OGNVs, how miR-18a induces the expression of IFNγ in macrophages required further investigated. We first searched miRNA databases for potential miR-18a targets that may possibly contribute to IFNγ induction. The three public miRNA databases (TargetScan, Pictar, and MicroRNA) all predicted that *Irf2* might be a target for miR-18a; the 3′-UTR of *Irf2* contains a highly conserved binding site from position 1668 to 1682 for miR-18a (Figure 6A). To determine whether miR-18a could target *Irf2* in macrophage cells, we transfected the mouse mature miR-18a mimic into BALB/c-derived macrophage-like RAW264.7 cells. The RAW264.7 cells transfected with OGNVs/miR-18a have significantly down-regulated IRF2 mRNA expression (Figure 6B) as well as IRF2 protein expression (Figure 6C, 6D). We also found IFNγ induction by OGNVs/miR-18a following reduction of *Irf2* (Figure 6C). *Irf2* siRNA repressed *Irf2* expression in RAW264.7 cells and led to increasing IFNγ expression (Figure 6E, 6F). These *in vitro* results were further confirmed in the liver KCs isolated from liver metastasis in CT26 mice administrated OGNVs/miR-18a (Figure 6G). To ascertain the direct effect of miR-18a on *Irf2*, a mutant construct that would disrupt the predicted miR-18a binding site was generated from pEZX-MT01- *Irf2* containing a 1,234 bp length 3′UTR of *Irf2* mRNA (Gene Accession: NM_008391.4). We performed a luciferase reporter assay by co-transfecting a vector containing IRF2 3′UTR fused luciferase and miR-18a or control miRNA as a negative control. Overexpression of miR-18a decreased the luciferase activity of the reporter with 3′UTR of *Irf2* by about 60% in RAW264.7 cells (Figure 6H). However, mutation that disrupted the binding site for miR-18a entirely restored luciferase activity. Moreover, overexpression of anti-sense (AS) miR-18a caused induction of luciferase and no inductive effect of AS-miR-18a on the activity of the reporter when a mutant 3′UTR of *Irf2* was detected. These results demonstrate that *Irf2* is a target of miR-18a in macrophages.

**Figure 7:**
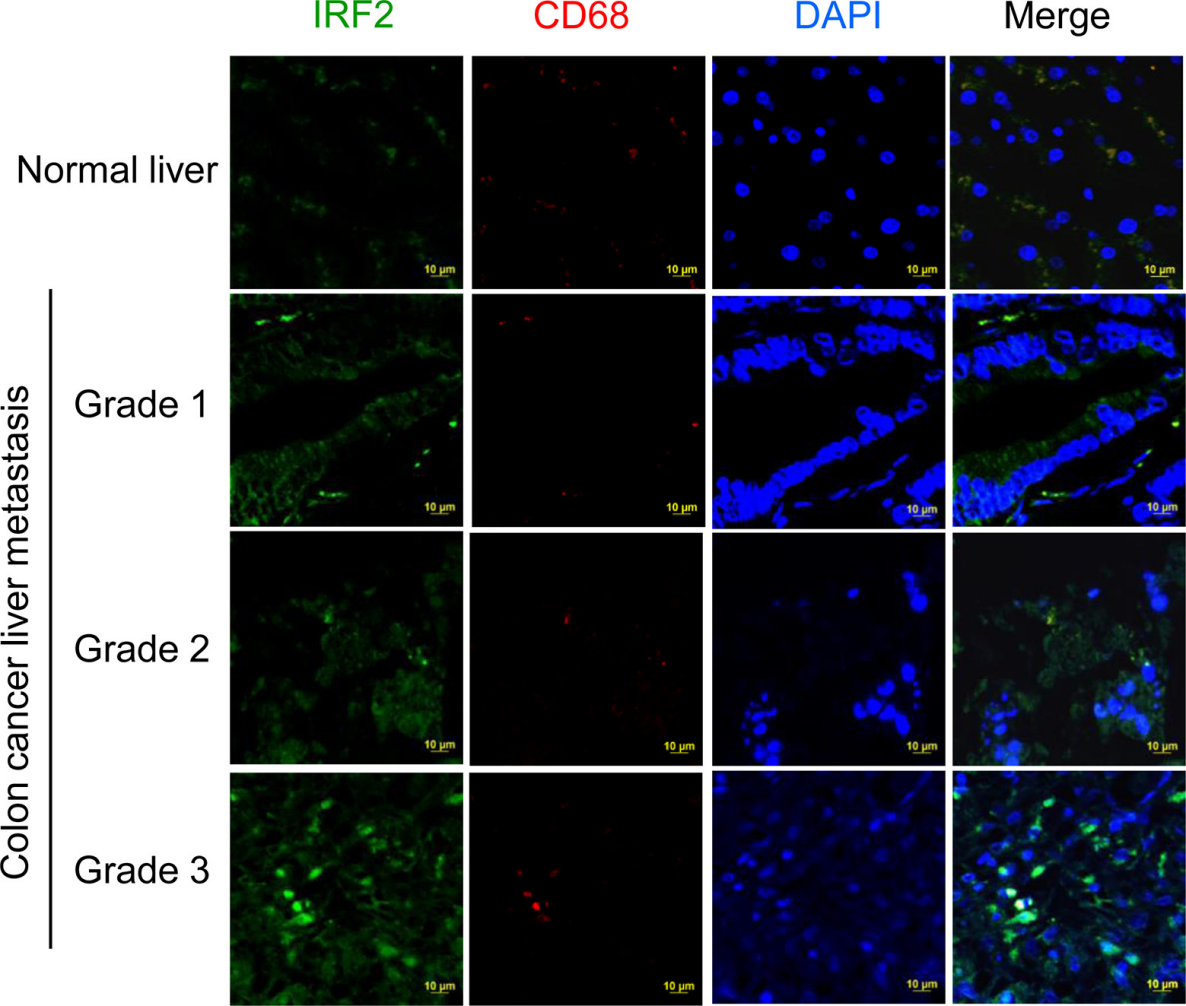
Up-regulation of IRF2 in metastatic liver tissue of colon cancer patients Double staining of human colon cancer tissue sections with antibodies against IRF2 (green) and against CD68 (red) followed by detection of fluorescence.

We further determined whether the *Irf2* was up-regulated in the metastatic liver tissue of colon cancer patients. The results from immunohistological staining of CD68 and IRF2 in human liver sections (Figure 7) suggest that IRF2 is expressed in liver CD68 macrophages. More importantly, the levels of expression of IRF2 in the liver of human colon metastatic patients are increased as the disease progresses. These results indicated that IRF2 expression correlates with liver metastasis differentiation in colorectal cancer.

## DISCUSSION

Liver metastases are common in many types of cancer, especially those of the gastrointestinal tract, breast, lung, and pancreas. Most treatments are not effective for liver metastasis because liver metastases represent cancer that has spread from another part of the body. We hypothesize that boosting the strength of anti-tumor immune responses may be a better way to treat liver metastasis; in particular, creating a liver microenvironment that is dominated by anti-tumor M1 macrophages.

In this study, our main finding is highlighted in a novel regulatory mechanism of M1 macrophage functioning along the IFN-γ/*Irf2* axis mediated by miR-18a (Supplementary Figure S7). These findings establish a proof of concept and the basis for treating liver metastasis of colon cancer by mediating macrophage populations which in turn could be applicable to other types of cancers and macrophage-mediated inflammatory diseases.

Liver macrophages are not only pleiotropic cells that can function as immune effectors and regulators, tissue remodelers, or scavengers [38], but also have unique location. KCs are stationary cells located in the vasculature, adherent to liver sinusoidal endothelial cells (LSECs) and directly exposed to the contents of blood. This is in contrast to other monocyte and macrophage cell populations located in other tissues that actively crawl through the tissue in search of pathogens or nano/micro particles. Importantly, the size of most nanoparticles, including GNVs, makes them favorable to being trapped in the liver. In addition, KCs represent 80–90% of all tissue macrophages in the entire body [39]. Collectively, these KCs features made GNVs favorable homing to the liver. The data presented in this study suggest that liver macrophages are preferentially targeted by GNV, and miR-18a delivered by GNVs to promote liver anti-tumor M1 macrophages induction. Since the liver is one of the major organs involved in metastasis for a number of different types of cancers, including colon cancer, and M1 macrophages play a role in an anti-tumor progression in general, our strategy could also be applied to treat other types of cancer with liver metastasis.

The acute inflammatory response is characterized by the presence of liver M1 macrophages, and the chronic or resolution of inflammatory phases is mediated by the enrichment of M2 macrophages. M1 macrophages are known to enhance anti-tumor growth and microbial clearance, and M2 macrophages are known to enhance liver tissue repair and to secrete pro-resolution substances including TGF-β. Therefore, targeted delivery of specific therapeutic agents which can modulate polarization of liver macrophages is critical. Our data presented in this study indicate that OGNVs are taken up by liver macrophages. The data we recently published [40, 41] and present in this study (Supplementary Figure S8) suggest that unlike commercially available vectors, OGNVs are non-toxic to the macrophages and liver and can be easily produced on a large scale basis for clinical applications and are capable of delivering a variety of different types of therapeutic agents.

In this study, we further optimized the conditions for OGNV delivery of mRNAs and miRNAs. Therefore, without manipulation of the OGNV, such as adding a targeting moiety, therapeutic agents delivered by OGNVs automatically get into liver macrophages with no toxic effects.

Different microRNAs are expressed in M1 or M2 macrophages and have been shown to control macrophage polarization. The role of miR-18a in macrophage polarization is unknown but immunomodulation of dendritic cell function of miR-18a has been described [42, 43]. We found that liver macrophages are polarized to M1 macrophages after miR-18a is delivered by OGNVs. The molecular mechanisms involved in miR-18a-induced M1 macrophages were further studied and we found that miR-18a-mediated induction of macrophage IFNγ is required for inhibition of liver metastasis of colon cancer and that macrophage IRF2 is targeted by miR-18a.

Unlike the situation with artificially synthesized nanoparticles, recently, we have developed grapefruit-derived nanovectors (GNVs) which can deliver a variety of therapeutic agents including chemotherapeutic compounds, DNA expression vectors, siRNA and proteins such as antibodies [41]. GNVs have a number of advantages over other delivery systems, including low toxicity, large scale production with low cost, and easily biodegradable without biohazards to the environment. However optimization of GNVs to maximize carrying therapeutic agents has not been studied. In this study, using miR-18a as an example, we found that optimized GNVs (OGNVs) are capable of encapsulating miR-18a and the ability was significantly increased by short pre-exposure of the GNVs mixed with miR-18a buffered with an optimized concentration of Na^+^ with exposure to ultraviolet (UV) light. We further demonstrate that miR-18a delivered by GNVs inhibits the growth of colon tumors that have metastasized to the liver by polarizing KCs to M1 cells (F4/80^+^IFNγ^+^IL-12^+^). miR-18a mediated induction of M1 IFNγ^+^ is required for production of IL-12. IL-12 subsequently triggers the activation of liver immune cells including NK and NKT cells. NOG mice lack mature T cells and functional NK cells. This role of IL-12 was also supported in NOG mice injected with CT26 colon tumor cells by the fact that miR-18a delivered by GNVs does not inhibit the growth of colon tumors that have metastasized to the liver. Nude mice which have both NK and NKT activity were found to inhibit the growth of metastasized tumors in the liver when injected with CT26 colon tumor cells. Although IL-12 has been shown to enhance the rejection of a variety of murine tumors, pre-clinical and clinical studies have revealed that IL-12 can produce severe toxicity [44]. Therefore, our finding that induction of IL-12 through KC IFN-γ induced through the GNV/miR-18a axis in the liver will have less side-effects compared to systemic administration IL-12 has great potential for anti-cancer immune therapy.

This study addresses the question of not only mechanisms that regulate the induction of M1 macrophages but also the use of grapefruit-derived nanovectors (GNVs) as a therapeutic vehicle for treatment of liver metastasis of colon cancer. We identified miR-18a as a previously unrecognized inhibitor for liver metastasis through the induction of M1 macrophage. These results provide new insights into the molecular mechanisms of miR-18a-mediated macrophage polarization and shed light on new therapies for cancers through a miR-18a-mediated induction of M1 macrophages. The means and method we demonstrated in this study are a major step in the development of high capacity GNVs to deliver therapeutic RNA in general.

Our findings established a basis for further investigating whether IRF2 acts as a suppressor to directly inhibit expression of IFNγ. Alternatively, it is possible that as a result of miR-18a-mediated down regulation of levels of IRF2, the level of IRF1 is increased. An imbalance between IRF-1 and IRF-2 [43, 44], the activator and repressor of IFN responses, respectively, may contribute to the altered expression of IFNγ. Therefore, increasing IRF-1/IRF-2 ratios by targeted delivery of miR-18a to IRF2 overexpressed macrophages is expected to induce IFNγ.

Systemic delivery of targeted vectors presents major challenges for developing an effective anti-cancer immunotherapy. One of advantages of an OGNV based delivering system is that OGNV is selectively taken up by liver KCs, not hepatocytes. Targeted delivery is particularly important for miRNA mediated therapy. One miRNA could regulate a number of genes, and among the potentially targeted genes, preferential miRNA targeted genes may be dependent on the levels of that miRNA and the accessibility and availability of the miRNA targeted genes. It is conceivable that the mRNA expression profile of one type of cell, such as KCs, targeted by OGNVs could be different from the hepatocytes. Therefore, genes targeted by miR-18a in KCs are unlikely the same ones if miR-18a is overexpressed in other types of cells such as hepatocytes. It has been reported that over expression of miR-18a in hepatocytes may contribute to the pathogenicity of liver cancer [45]. Our real-time PCR data showed that the level of miR-18a in hepatocytes was not increased following an intravenous administration of OGNVs/miR-18a. This could be due to OGNVs/miR-18a primarily being taken up by KCs. The exploitation of the liver macrophages to mediate the immune therapeutic effects of miRNA, such as miR-18a delivered by GNVs, can circumvent limitations of miRNA targeted delivery. Kupffer cells are the first point of contact to administer miRNAs encapsulated in OGNVs, affording an opportunity to directly modulate their functional activity. Therefore, besides of miRNAs, an OGNV based *in vivo* delivery system can also deliver other therapeutic agents which modulate liver macrophage activity and control macrophage lineage. OGNVs based targeting liver macrophage naturally take place without pressure on the host. Therefore, we do not expect that GNV based targeted delivery to KCs would be altered due host pressure built up as other delivery system.

## MATERIALS AND METHODS

### FISH (fluorescence *in situ* hybridization)

To visualize biotin conjugated miR-18a in the liver, tissue sections were deparaffinized and rehydrated. After permeabilization by adding 1% triton X-100, tissue sections were incubated in PBS containing 5 mg/ml of lysozyme at 37°C for 20 min. Following a pre-incubation at 46°C for 1 h in hybridization buffer (900 mM NaCl; 20 mM Tris-HCl, pH 8.0; 1 mM EDTA, pH 8.0), tissues were hybridized with 0.1 μM of Alexa Fluor^®^ fluorescent conjugated streptavidin at 46°C overnight. After dehydrating the tissue sections in a graded ethanol series, i.e., 70%, 80%, 95%, 100% ethanol, nuclear chromatin was stained with 4′, 6-diamidino-2-phenylindole (DAPI) and the tissues were analyzed using confocal laser scanning microscopy.

### Preparation and characterization of optimized GNVs (OGNVs)

Grapefruit-derived lipids were prepared, as previously described [41]. In brief, the sucrose gradient purified grapefruit nanoparticles were harvested from the 30%/45% interface (Supplementary Figure S1). The lipids were extracted with chloroform and dried under vacuum. The concentration of lipids was measured using the phosphate assay as described. To generate OGNVs, 200 nmol of lipid was suspended in 200–400 μl of 155 mM NaCl with 10 μg of RNA. After UV irradiation at 500 mJ/cm^2^ in a Spectrolinker (Spectronic Corp.) and bath sonication (FS60 bath sonicator, Fisher Scientific) for 30 min, the pelleted particles were collected by centrifugation at 100,000 g for 1 h at 4°C. The size and zeta potential distribution of the particles was analyzed using a Zetasizer Nano ZS (Malvern Instrument, UK).

### Labeling RNA in OGNV with Exo-GLOW

RNA in OGNVs was labeled with Exo-GLOW™ Exosome Labeling Kits (Cat # EXOR100A-1, System Biosciences) in accordance with the manufacturer's instructions. 10 μl of resuspended OGNVs with encapsulated RNA was diluted into 500 μl of PBS with 50 μl of 10x Exo-Red and incubated at 37°C for 10 min. To stop the labeling reaction, 100 μl of the ExoQuick-TC reagent was used and the reaction was placed on ice for 30 min. After washing by centrifugation at 13,000 rpm for 3 min, OGNVs were resuspended and were assessed for fluorescence intensity with an excitation maximum at 460 nm and emission maximum shift to 650 nm. Details of other methods used in this study are described in the Supplementary Experimental Procedures.

### Statistical analysis

Statistical significance was determined by the Student's *t* test. Differences between individual groups were analyzed by one- or two-way analysis of variance test. Differences were considered significantly when the *P* value was less than 0.05 or 0.01 as indicated in the text.

## SUPPLEMENTARY MATERIALS AND FIGURES


