# Supplementary material for: Grapefruit-derived nanovectors deliver miR-18a for treatment of liver metastasis of colon cancer by induction of M1 macrophages
Source: Oncotarget. 2016 Mar 25;7(18):25683–97. doi: 10.18632/oncotarget.8361 (PMC5041936; doi:10.18632/oncotarget.8361)
Supplement: Supplementary file 1 [file oncotarget-07-25683-s001.pdf]

Grapefruit-derived nanovectors deliver miR-18a for treatment of liver metastasis of colon cancer by induction of M1 macrophages

Supplementary Materials

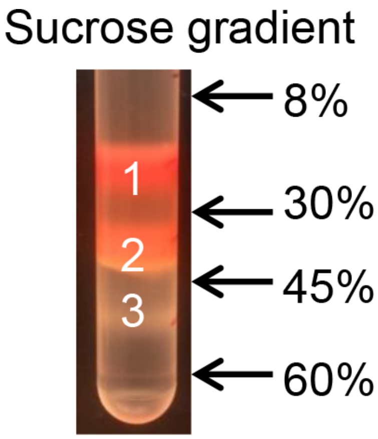

Supplementary Figure S1: Sucrose-banded particles from grapefruit juice. The nanoparticles were isolated from grapefruit juice using a sucrose gradient (8, 30, 45, and 60% sucrose in 20 mM Tri-Cl, pH 7.2). Particles from band 2 were used for preparation of OGNVs.

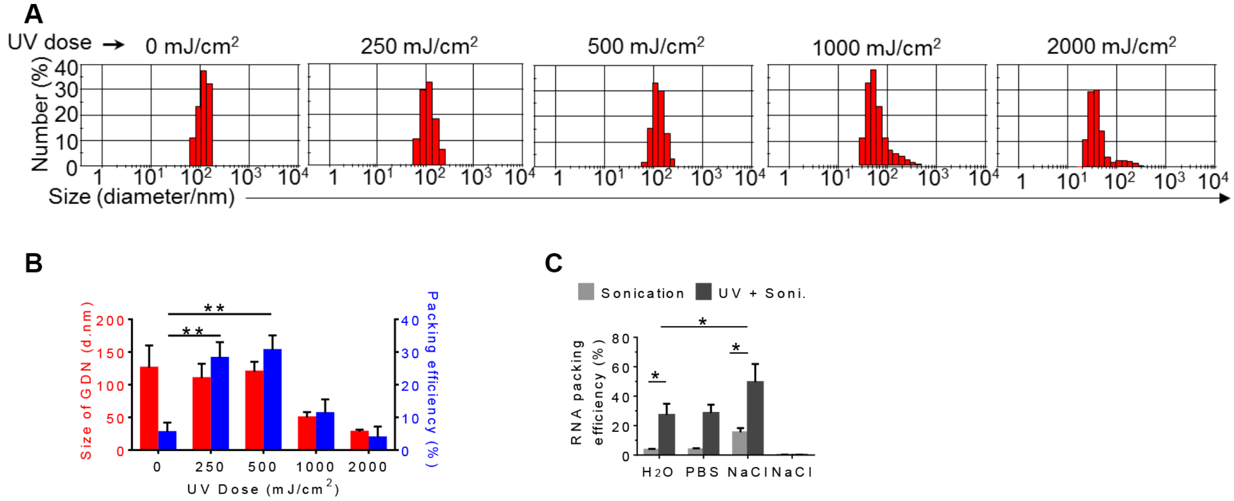

Supplementary Figure S2: The impact of optimization on the characteristics and biological activity of RNA packed in OGNVs. (**A**) Ultraviolet (UV) radiation at 0, 250, 500, 1000, 2000 millijoule per square centimeter (mJ/cm2) using a Spectrolinker prior to assembling OGNVs using grapefruit-derived lipids. Size distribution of OGNVs analyzed using a Zetasizer Nano ZS. (**B**) Quantification of OGNV packing efficiency (blue) and OGNV size distribution (red). OGNV packing efficiency was defined as the amount of RNA isolated from OGNVs divided by the amount of RNA initiated for use. (**C**) Evaluation of OGNV packing efficiency h prepared with H2O, PBS (pH 7.4), or NaCl (155 mM) prior to resembling OGNVs using grapefruit-derived lipids with sonication combining with UV (UV + Soni.) or not. *P < 0.05 and ***P* < 0.01 (two-tailed *t*-test). Data are representative of three independent experiments (error bars, S.E.M.).

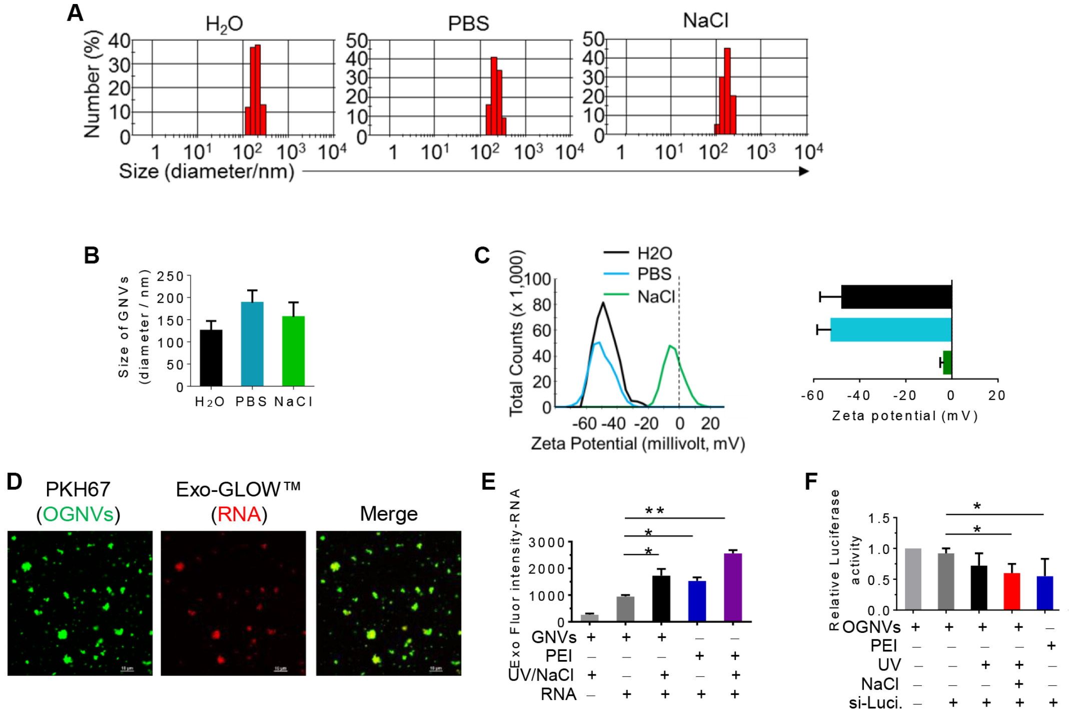

Supplementary Figure S3: The impact of optimization on the characteristics and biological activity of RNA packing in optimized OGNVs (OGNVs). (**A**) Size distribution of OGNVs analyzed using a Zetasizer Nano ZS. OGNVs prepared with H2O, PBS (pH 7.4), or NaCl (155 mM) prior to sonicate. (**B**) Quantification of OGNV size distribution prepared with H_2O, PBS (pH 7.4), or NaCl (155 mM). (**C**) Surface charge of OGNVs prepared with H_2O, PBS (pH 7.4), or NaCl (155 mM) and analyzed using a Zetasizer Nano ZS (left). Quantification of OGNV surface charge (right). (**D**) 200 mM of OGNVs prepared with 20 µg of total RNA and exposed to NaCl (155 mM) and UV (500 mJ/cm^2). Distribution of PKH67-labeled (green) OGNVs and Exo-GLOW-labeled (red) RNA visualized with confocal microscopy. (**E**) Fluorescence intensity of Exo-GLOW-labeled RNAs encapsulated in OGNVs measured by a Biotek Synergy HT plate reader (460 nm excitation, 420 nm emission). OGNVs prepared without RNA, with 20 µg of RNA only, with 20 µg of RNA and exposed to UV (500 mJ/cm2), polyethylenimine (PEI, 0.2 ng/µl), or a combination of UV and PEI. (**F**) Assessment of luciferase activity using the Dual-Luciferase Reporter Assay System (Promega) for the U87MG cells stably expressing firefly luciferase transfected with OGNVs or OGNVs encapsulating luciferase siRNA (si-Luci). *$P < 0.05$ and **$P < 0.01$ (twotailed *t*-test). Data are representative of three independent experiments (error bars, s.e.m.).

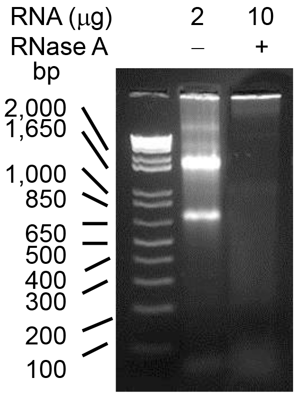

Supplementary Figure S4: OGNVs RNA is protected against Ribonuclease A (RNase A) digestion. OGNVs were treated with RNase A and then total RNA was extracted and digested with or without RNase A at 50 µg/ml. The samples were subsequently run on 1% agarose gel electrophoresis and gel was visualized by ethidium bromide staining.

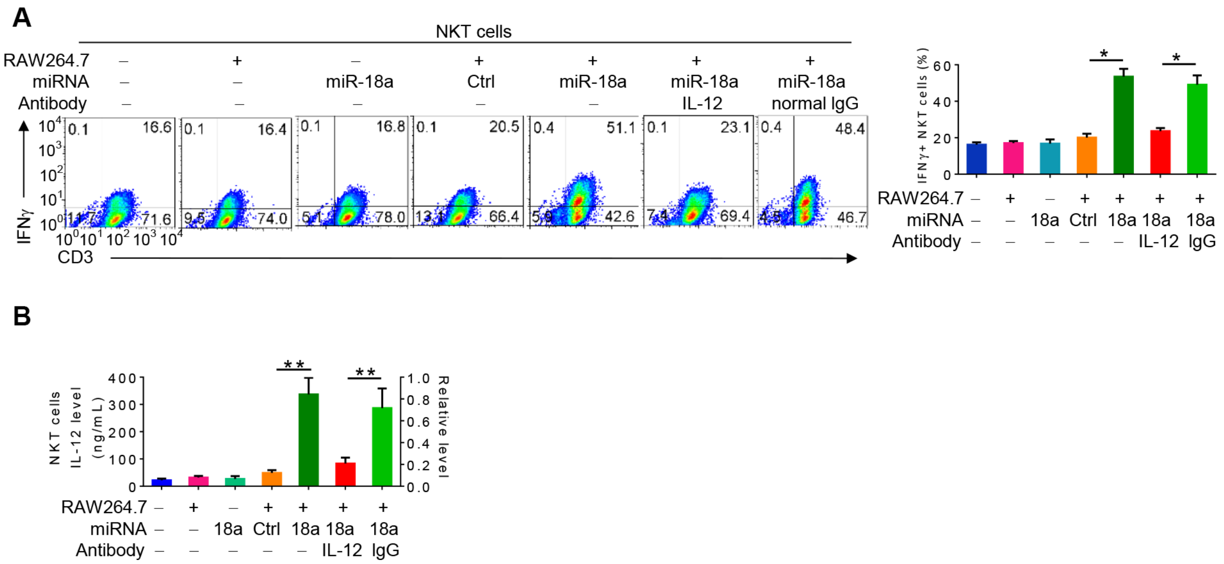

Supplementary Figure S5: (A) FACS sorted NKT cells were co-cultured with macrophage-like RAW264.7 cells pre-transfected with miR-18a in the presence of anti-IL-12 antibody or normal IgG as a control for 24 h and frequency of IFNγ+ NKT cells assessed by flow cytometry. Numbers in quadrants indicate percent cells in each; Right, quantification of FACS analyzed results. **P* < 0.05 (two-tailed *t*-test). Data are representative of three independent experiments (error bars, S.E.M.). (**B**) IL-12 level in the supernatants from experiment described above was quantitatively analyzed with ELISA. **P* < 0.01 (two-tailed *t*-test).

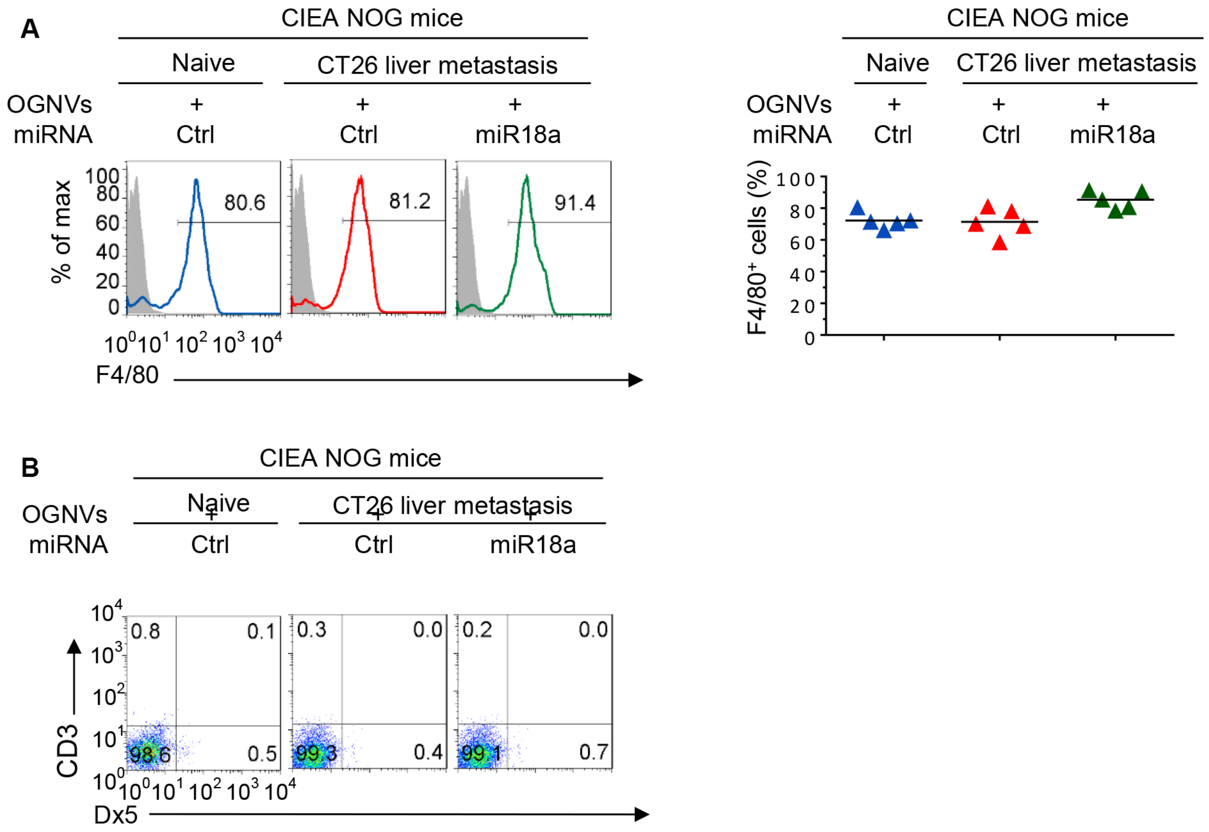

Supplementary Figure S6: NOG mice limit the response of miR-18a against liver metastasis. (**A**) Frequency of F4/80+ cells in liver leukocytes from NOG mice, CT26 liver metastasis treated with OOGNVs/Ctrl and OOGNVs/miR-18a mice assessed by flow cytometry. Numbers above bracketed lines and in quadrants indicate percent cells; gray, isotype-matched control antibody; Right, quantification of results at left. Each symbol represents an individual mouse. Data are representative of three independent experiments (error bars, S.E.M.). (**B**) Frequency of $CD3^+$, $Dx5^+$ cells in liver leukocytes cells from NOG, CT26 liver metastasis treated with OOGNVs/Ctrl and OOGNVs/miR-18a, mice assessed by flow cytometry. Numbers in quadrants indicate percent cells in each throughout.

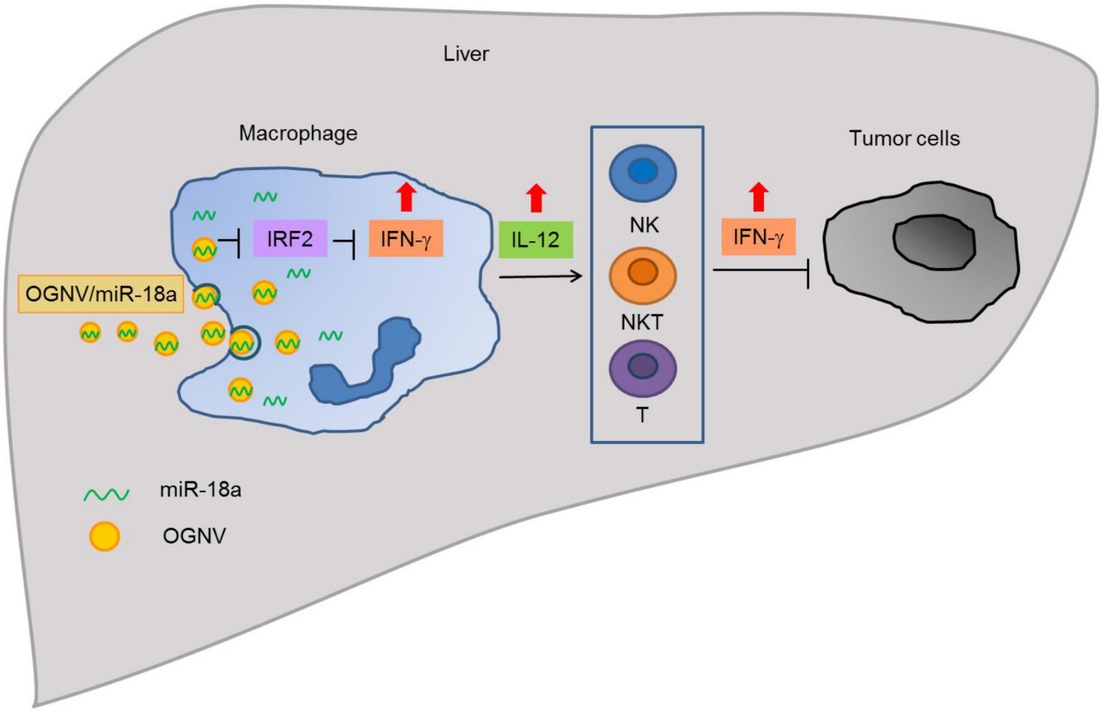

Supplementary Figure S7: Proposed pathway that leads to the induction of M1 macrophages mediated by miR-18a. miR-18a encapsulated in OGNVs (OGNVs/miR18a) is taken up by liver macrophages leading to down-regulation of IRF2. As a result of decreased IRF2, IFNγ is upregulated and subsequently stimulates the induction of M1 macrophages (F4/80+IL-12+) which further triggers anti-tumor activation of NK, NKT, and T cells.

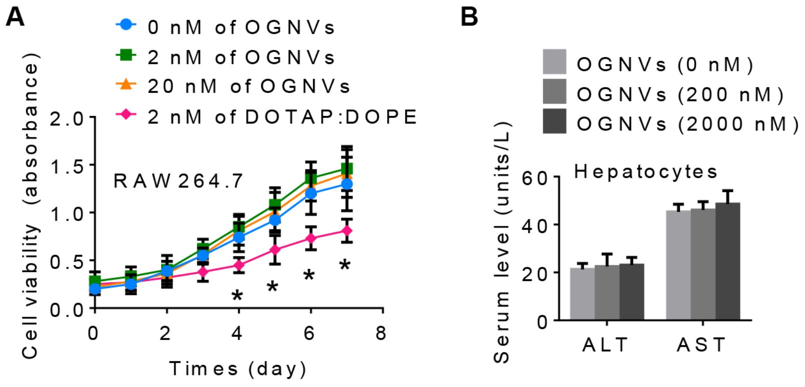

Supplementary Figure S8: No evidence for toxicity of the OGNVs to macrophages and hepatocytes. (**A**) Proliferation of cultured macrophage-like RAW264.7 cells treated with or without 2 nM or 20 nM OGNVs or 2 nM of DOTAP:DOPE liposomes. Cell viability was detected with the ATPlite assay from 0 d to 7 d after exposure to OGNVs or DOTAP:DOPE liposomes. (**B**) Quantitative evaluation of ALT and AST in OGNVstreated mice serum with Infinity ALT or AST Liquid Stable Reagent from Thermo Scientific. Data are representative of three independent experiments (error bars, s.e.m.). $^{*}P < 0.05$ (two-tailed *t*-test).

SUPPLEMENTAL PROCEDURES

Mouse model study

8– to 12– week-old female BALB/C mice, Interferon γ(IFNγ) knockout mice, athymic immunodeficient nude mice and Central Institute for Experimental Animals (CIEA) NOG (NOD/Shi-scid,IL-2RYnull) mice. The NOG mice lack mature T cells, B cells, functional NK cells, and are also deficient in cytokine signaling. The athymic immunodeficient nude mice lack T cells but have NK activity. All mice were purchased from The Jackson Laboratory (Bar Harbor, ME) and housed under specific pathogen-free conditions. Animal care was performed following the Institute for Laboratory Animal Research (ILAR) guidelines and all animal experiments were done in accordance with protocols approved by the University of Louisville Institutional Animal Care and Use Committee (Louisville, KY). The mice were acclimated for at least 1 week before any experiments were conducted.

For animal model of colon cancer with liver metastasis, mice were anaesthetized with a mixture of ketamine and xylazine. 1×10^6 of CT26 colon cancer cells were administered via intra-splenic injection as previously described(Jiang, Wang et al., 2014). The treatment schedule was arranged as following: thirty 7-week old male BALB/c mice were randomly divided into two groups, with 15 mice per group. The first group of mice (n = 15) was treated as following: 10/15 of mice were intra-spenic injected with CT26 tumor cells at day 0 and 5/15 mice were injected with PBS as a control. The second group of mice (n = 15) were housed in the same environment as first group mice until day 12. At day 12, 10/15 of the second group of mice were intra-splenic injected with CT26 tumor cells and 5/15 mice were injected with PBS as a control. For both groups of mice, day 2 after intra-splenic injection 200 nM OGNVs packed with 2 nM of miR-18 (OGNVs/miR18a) or Scramble miRNA (OGNVs/Ctrl) as a control were administrated to mice via tail veil injection. For the first group of mice, mice were treated three times per week for 2 weeks. For the second group of mice, the mice were treated one time on day 2 after CT26 injection. On day 14 mice were sacrificed and various organs were removed for examination. The percentages of NK, NKT, and T cells isolated from each group of treated mice were also FACS analyzed.

Liver macrophage depletion

Mice were injected with approximately 110 mg/kg of clodronate liposomes (FormuMax Scientific Inc.) i.p. or an equal volume of PBS liposomes. The injection was repeated three days later and experiments were performed 4 days after the first injection.

Antibodies and reagents

The following monoclonal antibodies (eBioscience) were used for flow cytometry: F4/80 (17-480182), anti-CD3 (46-0032-82), anti-Dx5 (17-5971-82), anti-IL-12 (12-7123-82), anti-CD80 (12-080182), anti-CD86 (11-0862-85), anti-IFN-γ (11-7311-82). The following monoclonal antibodies purchased from Biolegend were used for flow cytometry: anti-CD3 (100206), anti-Dx5 (103503), anti- anti-MHCII (107624), anti-IL-12 (505205), anti-CD80 (122007), and anti-CD86 (105027). The anti-mouse IL-12 (p70) antibody (511802, clone C18.2) purchased from Biolegend was used for neutralization of IL-12.

Cell culture

The BALB/c syngeneic CT26, undifferentiated colon cancer cell line, and RAW264.7, murine macrophage cell line (American Type Culture Collection, Rockville, MD) were grown at 37°C in 5% CO_2 in Dulbecco's Modified Eagle's medium (DMEM) medium and RPMI 1640 medium (Gibco), respectively, supplemented with 10% heat-inactivated fetal bovine serum (FBS), 100 U/ mL penicillin, and 100 ^g/mL streptomycin.

Flow cytometry

Liver and spleen from mice were removed and gently pressed through nylon cell strainers (70 μm in diameter, Fisher Scientific) to obtain single-cell suspensions in RPMI-1640 containing 5% FBS. Hepatocytes were removed from liver-cell suspensions by colloidal silica particle (Percoll, Invitrogen) gradient centrifugation in phosphate-buffered saline. Erythrocytes in liver and spleencell suspensions were then removed using ammonium-chloride-potassium (ACK) lysing buffer (0.15 M NH4Cl, 10 mM $KHCO_3$, 0.1 mM EDTA). Washed cells were stained for 40 min at 4°C with the appropriate fluorochrome-conjugated antibodies in PBS with 2% FBS. To detect intracellular antigens, washed cells were incubated in diluted Fixation/ Permeabilization solution (eBioscience Cat# 00-5123) at 4°C for 30 min. Characterization and phenotyping of the various lymphocytes subsets from liver or spleen were performed by flow cytometry. Data were acquired on BD FACS Canto (BD Biosciences, San Jose, CA) and were analyzed using FlowJo software (Tree Star Inc., Ashland, OR). Numbers above bracketed lines in FACS figures indicate percent of positive stained cells, and the results of cells stained with a isotype-matched control antibody are shown in gray color.

Intracellular cytokine production

Lymphocyte preparations were stimulated for 6 h with PMA (phorbol 12-myristate 13-acetate; 1 ng/ ml; Invitrogen) and ionomycin (1 ^M; Invitrogen), LPS (10 µg/ml), or GalGer (100ng/ml) in the presence of brefeldin A (5 µg/ml; Invitrogen). Cells were then stained for markers of NKT cells, NK cells, and T cells with anti-CD3 and anti-Dx5. The cells were fixed and permeabilized with fixation and permeabilization buffers (BD Biosciences) and intracellular IL-12, IFN-γ and TGFp were stained and FACS-analyzed.

Site-directed mutagenesis within the IRF2 promoter

We utilized two algorithms that predict the mRNA targets of miRNAs, TargetScan (http://www.targetscan.org) and microRNA (http://www.microRNA.org), and Pictar (http://http://pictar.mdc-berlin.de/. IRF2 was selected by both online tools with strong conserved 3′untranslated region (3′UTR) sites. To determine the ability of miR-18a to target the 3′UTR- IRF2 activity, a luciferase reporter containing 1,234 bp of the IRF2 3′UTR in the pEZX-MT01 vector was purchased from GeneCopoeia (Cat# MmiT027452-MT01, Rockville, MD). The mutant of IRF2 3′UTR was generated with the oligonucleotide primer IRF2-Mut, which was designed to specifically disrupt putative IRF2 at its 3′UTR site. Q5® Site-Directed Mutagenesis Kit (New England Biolabs, MA, USA) was used in conjunction with specific primers (Supplementary Table 1) to introduce IRF2 3′UTR mutations in the pEZX-MT01 construct according to the manufacturer's instructions. After mutant strand synthesis and ligation, resultant plasmids were introduced into *E. coli* and transformants were selected using kanamycin resistance. Further DNA sequence of mutant was confirmed by DNA sequencing.

Transient transfection and luciferase reporter assay

Murine macrophage RAW264.7 cells were plated in 24-well plates at a density of $3.0*10^4$ cells/well in antibiotic free RPMI-1640 medium supplemented with 10% FBS. 100 ng of pEZX- MT01 or mutant luciferase reporter were transfected using FuGENE HD Transfection Reagent (Roche Applied Science, Indianapolis, IN) with 10 pmol of mimic mmu-miR-18a and Opti-MEM® Reduced Serum Medium (Invitrogen, Carlsbad, CA). For all reporter assays, the cells were harvested 48 h post-transfection using Promega's Passive Lysis buffer. The activities of luciferase in cell lysates were determined using the Dual-Luciferase Reporter Assay System (Promega). Relative expression (fold change) was determined by dividing the averaged normalized values from mock transfection. Values were averaged as indicated in the Figure legends.

Labeling OGNVs with PKH67

OGNVs were labeled with PKH67 Fluorescent Cell Linker Kits (Sigma) in accordance with the manufacturer's instructions. OGNVs were suspended in 250 µl of Diluent C with 1 µl of PKH67 and mixed with 250 µl of dye solution for subsequent incubated with an equal volume of 1% BSA for 1 min at 22°C. After centrifugation for 5 minutes at 13,000 rpm, 20 µl of resuspended labeled OGNVs were loaded on a slide for assessment of viability using confocal microscopy (Nikon).

Quantitative Real-Time PCR (qPCR) analysis of miRNA and mRNA expression

Total RNA was isolated from lymphocyte cells with a miRNeasy mini kit (Qiagen) and reverse- transcribed using a miRNA reverse transcription kit (Qiagen). Mature miR-18a expression was quantified by quantitative real-time PCR (qPCR) using a miScript II RT kit (Qiagen) and miScript SYBR Green PCR Kit (Qiagen) with Qiagen predesigned primers. All kits were used according to the manufacturer's instructions. U6 transcript was used as an internal control to normalize RNA input. For analysis of IL-12, IFNy, MHCII, TGFp, IRF1,IRF2, Smad2, ESR1, ESR2 mRNA expression, 1 µg of total RNA was reverse transcribed by SuperScript III reverse transcriptase (Invitrogen) and quantitation was performed using primers (Eurofins) with SsoAdvancedTM Universal SYBR Green Supermix (BioRad) and p-actin was used for normalization. The primer sequences are listed in Supplementary table 1. qPCR was run using BioRad CFX96 qPCR System with each reaction run in triplicate. Analysis and fold change were determined using the comparative threshold cycle (Ct) method. The change in miRNA or mRNA expression was calculated as fold-change.

Western blotting

Cells were treated as indicated in individual Figure legends and whole cell extracts (WCE) were prepared in modified RIPA buffer (Sigma) with addition of protease and phosphatase inhibitors (Roche). Western analysis was performed and quantitated as described (Jiang et al., 2014).

Proteins were separated by 10% SDS-PAGE and transferred to PVDF membranes (Bio-Rad Laboratories, Inc., Hercules, CA). Dual color precision protein MW markers (BioRad) were separated in parallel. Antibodies were purchased as follows: IRF2 (sc-498), a-tubulin (sc-8035), from Santa Cruz Biotechnology (Santa Cruz, CA) and IFNy (ab9657, Abcam). The secondary antibodies conjugated to Fluors Alex-488 or Alex-594 were purchased from Invitrogen (Eugene, OR). The bands were visualized on the Odyssey Imager (LiCor Inc, Lincoln, NE).

Histological analysis

Tissues were fixed with buffered 10% formalin solution (SF93-20; Fisher Scientific, Fair Lawn, NJ) overnight at 4°C. Dehydration is achieved by immersion in a graded ethanol series, 70%, 80%, 95%, 100% ethanol for 40 min each. Tissues were embedded in paraffin and subsequently cut into ultra-thin slices (5 um) using a microtome. Tissue sections were stained with hematoxylin and eosin, and slides were scanned with an Aperio ScanScope. For frozen sections, tissues were fixed with periodate-lysine-paraformaldehyde (PLP) and dehydrated with 30% sucrose in PBS at 4°C, overnight. Tissue sections were stained with primary Ab in PBS/5% BSA (1:200) for 2 h and secondary Ab in PBS/5% BSA (1:800) for 30 min. 4′,6-Diamidino-. 2-phenylindole dihydrochloride (DAPI) was used for nuclear stain. Human colon cancer tissues slides, metastatic tissue and adjacent normal tissue were purchased from US Biomax Inc (Rockville, MD, Cat# CO702).

Enzyme-linked immunosorbent assay (ELISA)

The cytokine IL-12 in culture supernatants was quantified using ELISA kits (eBioscience) according to the manufacturer's instructions. Briefly, a microtiter plate was coated with anti-mouse IL-12 antibody at 1:200 overnight at 4°C. Excess binding sites were blocked with 200 µl of 1x ELISA/ELISPoT Diluent (eBioscience) for 1 h at 22°C. After washing three times with PBS containing 0.05% Tween 20, the plate was incubated with detective antibody in blocking buffer for 1 h at 22°C. After wash three times, the Avidin conjugated with horseradish peroxidase and substrate were presented for 1 h and 30 min at room temperature respectively, followed an analysis of absorbance at 405 nm using a microtiter plate reader (BioTek Synergy HT).

REFERENCES

1. Jiang H, Wang P, Li X, Wang Q, Deng ZB, Zhuang X, Mu J, Zhang L, Wang B, Yan J, Miller D, Zhang HG. Restoration of miR17/20a in solid tumor cells enhances the natural killer cell antitumor activity by targeting Mekk2. Cancer immunology research. 2014; 2:789–99
